# Supplementary material for: Brain morphometry and chronic inflammation in Bangladeshi children growing up in extreme poverty
Source: Imaging Neurosci (Camb). 2024 Oct 16;2:imag-2-00319. doi: 10.1162/imag_a_00319 (PMC12290609; doi:10.1162/imag_a_00319)
Supplement: Supplementary Material [file imag_a_00319-supp.pdf]

Supplementary Tables

Supplementary Table 1. Descriptive statistics for concentrations of C-reactive protein

Timepoint (weeks)	Median (mg/L)	Minimum (mg/L)	Maximum (mg/L)
6	0.085	0	7.37
18	0.5	0	33.42
40	1.725	0	72.58
53	0.82	0	28.88
104	1.2	0	62.87
156	0.55	0.4	110.05
207	0.58	0.04	58.34
260	1.365	0	57.59

Supplementary Table 2. Associations between basal ganglia volumes and inflammation across developmental time points

Brain Areas	Week 6	Week 18	Week 40	Week 53	Week 104	Week 156	Week 207	Week 260
L. Caudate	-0.19	-0.18	-0.15	-0.12	-0.20	-0.16	-0.12	-0.07
L. Putamen	0.00	**-0.24**	**-0.29**	**-0.26**	-0.07	-0.13	-0.15	0.03
L. Pallidum	-0.10	-0.13	-0.19	**-0.26**	-0.21	-0.07	0.04	0.09
R. Caudate	-0.18	-0.17	-0.15	-0.17	-0.16	-0.15	-0.12	-0.05
R. Putamen	0.02	-0.20	**-0.32**	**-0.30**	-0.06	-0.08	-0.18	-0.11
R. Pallidum	-0.09	**-0.27**	-0.21	**-0.23**	-0.08	-0.14	-0.04	0.21

Bold indicates $p < 0.05$

Supplementary Table 3. Associations between basal ganglia volumes and average inflammation across developmental time points

Brain Areas	r	p
L. Caudate	-0.23	4.4E-02
L. Putamen	-0.22	5.7E-02
L. Pallidum	-0.19	9.7E-02
R. Caudate	-0.24	3.2E-02
R. Putamen	-0.28	1.2E-02
R. Pallidum	-0.19	1.0E-01

Supplementary Table 4. Associations between basal ganglia volumes and chronic inflammation with elevations measured as [CRP] > 1 mg/L

Brain Areas	r	p
L. Caudate	-0.24	3.3E-02
L. Putamen	-0.37	8.6E-04
L. Pallidum	-0.29	8.9E-03
R. Caudate	-0.29	9.9E-03
R. Putamen	-0.42	1.1E-04
R. Pallidum	-0.30	7.3E-03

Supplementary Figures

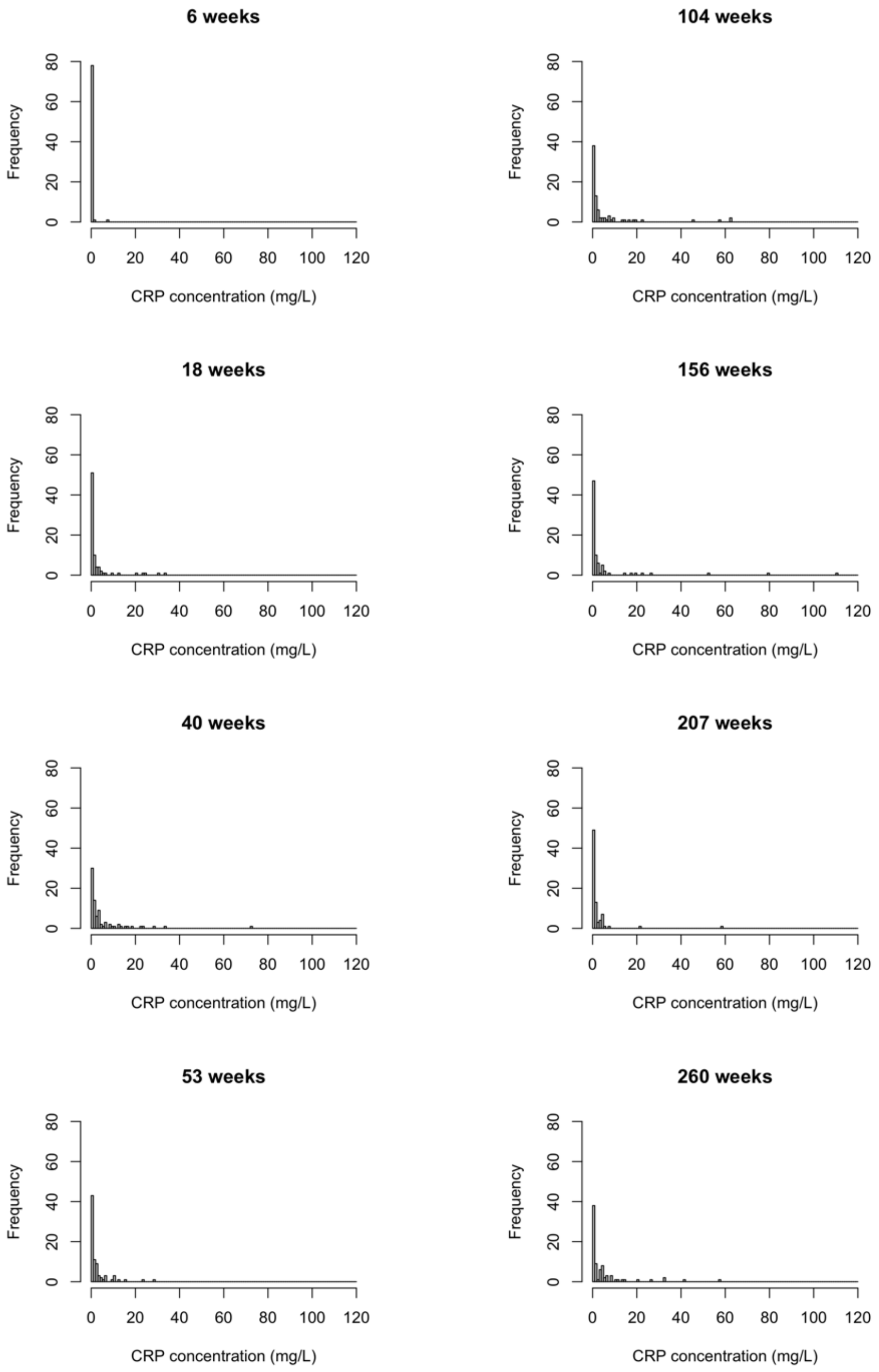

Supplementary Figure 1. Distribution of C-reactive protein (CRP) concentrations. Histograms depict the distribution of CRP concentrations across eight developmental time points.
